# IFN-β mediates the anti-osteoclastic effect of bisphosphonates and dexamethasone

**DOI:** 10.3389/fphar.2022.1002550

**Published:** 2022-10-14

**Authors:** Prajakta Kalkar, Gal Cohen, Tal Tamari, Sagie Schif-Zuck, Hadar Zigdon-Giladi, Amiram Ariel

**Affiliations:** ^1^ Departments of Biology and Human Biology, University of Haifa, Haifa, Israel; ^2^ Laboratory for Bone Repair, Rambam Health Care Campus, Haifa, Israel; ^3^ The Ruth and Bruce Rappaport Faculty of Medicine, Technion—Israel Institute of Technology, Haifa, Israel

**Keywords:** IFN-β, resolution of inflammation, macrophages, osteoclast differentiation, dexamethasone, zoledronic acid, multiple myeloma

## Abstract

Zoledronic acid (Zol) is a potent bisphosphonate that inhibits the differentiation of monocytes into osteoclasts. It is often used in combination with dexamethasone (Dex), a glucocorticoid that promotes the resolution of inflammation, to treat malignant diseases, such as multiple myeloma. This treatment can result in bone pathologies, namely medication related osteonecrosis of the jaw, with a poor understanding of the molecular mechanism on monocyte differentiation. IFN-β is a pro-resolving cytokine well-known as an osteoclast differentiation inhibitor. Here, we explored whether Zol and/or Dex regulate macrophage osteoclastic differentiation *via* IFN-β. RAW 264.7 and peritoneal macrophages were treated with Zol and/or Dex for 4–24 h, and IFN-β secretion was examined by ELISA, while the IFN stimulated gene (ISG) 15 expression was evaluated by Western blotting. RANKL-induced osteoclastogenesis of RAW 264.7 cells was determined by TRAP staining following treatment with Zol+Dex or IFN-β and anti-IFN-β antibodies. We found only the combination of Zol and Dex increased IFN-β secretion by RAW 264.7 macrophages at 4 h and, correspondingly, ISG15 expression in these cells at 24 h. Moreover, Zol+Dex blocked osteoclast differentiation to a similar extent as recombinant IFN-β. Neutralizing anti-IFN-β antibodies reversed the effect of Zol+Dex on ISG15 expression and partially recovered osteoclastic differentiation induced by each drug alone or in combination. Finally, we found Zol+Dex also induced IFN-β expression in peritoneal resolution phase macrophages, suggesting these drugs might be used to enhance the resolution of acute inflammation. Altogether, our findings suggest Zol+Dex block the differentiation of osteoclasts through the expression of IFN-β. Revealing the molecular pathway behind this regulation may lead to the development of IFN-β-based therapy to inhibit osteoclastogenesis in multiple myeloma patients.

## 1 Introduction

Immune cells and cytokines are critical effectors in bone remodeling during inflammation and its resolution, as well as in cancer-associated osteopathologies (Tai et al., 2018; Alvarez et al., 2019; Plemmenos et al., 2020). Zoledronic acid (Zol), a nitrogen-containing bisphosphonate (BP), together with the glucocorticoid dexamethasone (Dex), is commonly used for the treatment of MM (Ishikawa et al., 1990). The beneficial action of Zol in MM is mostly attributed to the induction of osteoclast death that limits the formation of lytic lesions (Takayanagi et al., 2002; Lee and Kim, 2011; Schett, 2011). At the cellular level, Zol is taken up by ostoclasts and inhibits the enzyme farnesyl diphosphate synthase. As a result, there is a reduction in cholesterol synthesis, which is required for cytoskeletal reorganization and vesicular trafficking in the osteoclast, leading to osteoclast inactivation (Reszka and Rodan, 2003). The mechanism of action of Dex in MM is not completely elucidated. Dex reduces IL-6 mRNA levels in myeloma cells and induces plasma cell apoptosis by blocking IL-6 (Alexanian et al., 1992). The combined effect of Zol and Dex on osteoclast formation has not been extensively studied**.** Nevertheless, clinical evidence showed this drug combination increase the risk for a severe side effect called medication-related osteonecrosis of the jaw (MRONJ) (Hüni and Fryar, 1981). MRONJ is characterized by formation of a necrotic jawbone usually after tooth extraction, in patients taking antiresorptive drugs, like BPs, or anti-receptor activator of nuclear factor kappa-B ligand (RANK-L) antibodies alone or in combination with immune modulators or anti-angiogenic medications (Ruggiero et al., 2022).

The interplay between immune cells and osteoclasts was previously reported. Immune cells secrete pro and anti-inflammatory cytokines that balance bone resorption and apposition (Roodman, 1993; Van Dyke et al., 2015). Cytokines that stimulate bone resorption include IL-1, TNF-α, IL-6, IL-11, IL-15, and IL-17. Inhibitors of resorption include IL-4, IL-10, IL-13, IL-18, GM-CSF, and IFN-γ. TGF-β and prostaglandins can have either stimulatory or inhibitory effects on resorption, depending on the experimental setting (Martin et al., 1998). The role of cytokines in hematological malignancies, including MM, revealed dysregulation of various cytokines that uncouple the balance between bone resorption and bone apposition, leading to the development of lytic bone lesions (Guise and Mundy, 1998).

Interferon *β* (IFN-β) belongs to the type 1 interferon (IFN) family, representing the first line of endogenous defense mechanisms in response to viruses and bacterial infections. These cytokines are secreted by many cell types, including lymphocytes, macrophages, and endothelial cells (Pertsovskaya et al., 2013). IFN-β promotes bacterial clearance, neutrophil apoptosis, and efferocytosis, as well as macrophage reprogramming to resolution-promoting phenotypes (Kumaran Satyanarayanan et al., 2019). IFN-β is produced in response to M-CSF stimulation of macrophage progenitors as part of the osteogenic process (Yamashita et al., 2012). Similarly, RANKL induces the production of IFN-β in macrophages during osteoclast differentiation. Interestingly, recombinant mouse IFN-β strongly inhibits osteoclastogenesis from bone marrow macrophages stimulated by RANKL in the presence of M-CSF. These results suggest that IFN-β interferes with RANKL signaling, thereby inhibiting osteoclastogenesis (Stark et al., 1998).

The combined therapy of Zol+Dex delays the progression or occurrence of bone lesions in MM patients (Tosi et al., 2006). We hypothesized that this drug combination increases IFN-β expression and secretion in macrophages, thereby reducing osteoclastogenesis. The current study aimed to improve our understanding of the molecular mechanism executed by Zol and Dex in the blocking of osteoclastogenesis, focusing on IFN-β. Revealing the aforementioned molecular pathway may perpetuate the development of new biological treatments to inhibit osteoclastogenesis and prevent the worsening of osteolytic lesions following chemotherapy.

## 2 Methods

### 2.1 Cell culture

RAW 264.7 macrophage cells (ATCC, TIB-71, Virginia) were cultured in Minimum Essential Medium-α (MEM-alpha, Biological Industries, Israel) containing 10% fetal bovine serum (FBS, Biological Industries, Israel), 100 μg/ml penicillin and streptomycin (Biological Industries, Israel) at 37°C in a humidified atmosphere of 5% CO_2_. The culture medium was changed every 3 days. Cells (1.5 × 10^6^ cells) were seeded in a small flask (25 cm^2^, Corning, Israel) for expansion for 3 days, and transferred to a big flask (175 cm^2^, Corning, Israel) with culture medium.

#### 2.1.1 Isolation of peritoneal macrophages

Male C57BL/6 mice were injected intraperitoneally with freshly prepared zymosan A in PBS (1 mg/ml/mouse). After 66 h, the peritoneal exudates were collected. Macrophages were labeled with PE-conjugated rat anti-F4/80 and isolated using EasySep PE selection magnetic beads following the manufacturer’s instructions (Stem-Cell Technology). All animal experiments were approved by the ethics committee for animal experimentation at the University of Haifa (no 597/18).

### 2.2 RT-PCR

Peritoneal macrophages (1*10^6^ cells per ml per treatment) were treated with Zol and/or Dex (5–10 µM and 1–10 µM, respectively, as in (Ural et al., 2003) in RPMI, respectively, for 4 h or 24 h. RNA extraction and cDNA synthesis were performed (Applied BioSystem, California). Then, qPCR was performed in triplicates using specific primers for IFN-β. IFN-γ and IFN-α were analyzed as reference genes and HPRT as a housekeeping gene. The reactions were normalized to mHPRT using the ΔΔ threshold cycle (Ct) method. Mouse primer sequences were as follows: mHPRT- Forward 5′- TTG​CTC​GAG​ATG​TCA​TGA​AGG​A -3′, and Reverse 5′- AGCAGGTCAGCAAA GAACTTATAGC -3′, m-IFN-γ: Forward:5′-GCGTCATTGAATCACACCTG-3′ and Reverse:5′- TGAGCTCATTGAATG CTTGG-3′, m-IFN-α-Forward:5′-CCTGAGAGA GAAGAAACACAGCC-3′ and Reverse: 5′-TCTGCT CTGACCACTCCCAG -3′, mIFN-β-Forward:5′-AACCTCACAGGGCGGACTT-3′ and Reverse: TCC​CAC​GTC​AAT​CTT​TCC​TCT​TG-3′ (Sigma Aldrich, Israel). Quantitative RT-PCR analysis was performed using a SyberGreen system on a Step One Plus (Thermo Fisher, Israel).

### 2.3 Western blotting

The expression of IFN-β, ISG15, or GAPDH proteins by macrophages (peritoneal or RAW 264.7) treated with vehicle, Zol, Dex, or Zol+Dex (1.5*10^6^ cells per ml per treatment, 4 or 24 h) was determined. To this end, the protein content of lysed cells was extracted and run using 10% SDS-PAGE (40 µg/lane). Next, separated proteins were transferred to nitrocellulose or PVDF membranes and immunoblotted with rabbit anti-IFNβ, mouse anti-ISG15, or rabbit anti-GAPDH, respectively (Santa-Cruz Biotechnology). The membranes were washed and incubated with appropriate HRP-conjugated secondary antibodies. Then, the membranes were washed, developed using WesternBright ECL (Advansta, CA), and analyzed using Amersham Imager 600. Our analysis focused on the high molecular weight isoforms of IFN-β that are non-secreted intracellular proteins (higher molecular weight than 33 kDa), while the secreted forms (25–33 kDa) were excluded**.** Densitometry analysis was performed using the ImageJ software.

### 2.4IFN-β ELISA

Culture media from macrophages treated with Zol and/or Dex or vehicle for 4 h were collected and evaluated for their IFN-β content by custom-made ELISA as in. Briefly, MaxiSorp plates were coated with purified anti-mouse IFNβ capture antibody (1 mg/ml) (BioLegend 519202) and incubated overnight at 4°C. Plates were washed 4 times with 0.05% PBS-Tween-20 and blocked at room temperature for 1 h with 1% B.S.A. in PBS. Plates were washed 4 times before 100 µl of standard (BioLegend 581309), or culture supernatants were plated in duplicate and incubated overnight at 4°C. Plates were washed 4 times and incubated with biotinylated anti-IFN-β detection antibody (BioLegend 508105) at 1 mg/ml at room temperature for 1 h. Plates were washed 5 times and incubated with HRP-Avidin for 30 min at room temperature and then developed using TMB substrate and stopped using 2 N sulfuric acid. Plates were read using BioTek PowerWave Plate reader at 450 nm and 540 nm. Results were calculated using a 4-parameter curve-fitting with Gen5 software (BioTek).

### 2.5 *In vitro* differentiation of macrophages to osteoclasts

Osteoclastogenesis assay was performed with RAW 264.7 cells (1.5*10^4^ cells per well in a 24-well plate) that were incubated with 30 ng/ml RANKL (Peprotech, Israel) for 5 days. RANKL-treated cells were also treated with Zol and/or Dex, recombinant mouse IFN-β (0.25 or 2.5 ng/ml, Biolegend), or anti-IFN-β antibodies (2 μg/ml, Abcam, United Kingdom) for the first 2 days of incubation and then washed. RANKL was supplemented after washing.

### 2.6 TRAP and CD11b staining

To characterize RAW 264.7 cells after differentiation, TRAP and immune-staining were performed. The cells were fixed with 4% paraformaldehyde (PFA)/PBS for 10 min at R.T. Immunocytology was used to detect cells that differentiated into osteoclasts (TRAP^+^CD11b^−^ cells). The cells were stained with a TRAP kit (387A-1KT, Sigma, United States) for 1 h at 37°C. Then, cells displaying deep purple staining (indicating high TRAP staining) were enumerated as cells that differentiated into osteoclasts. In addition, the cells were stained with anti-CD11b (ab52478, Abcam, United Kingdom) to indicate non-differentiated macrophages. The staining was performed as follows: After fixation, the cells were blocked with 1% BSA for 1 h, washed 3 times with PBS, and stained with Rabbit anti-CD11b for 1 h at R.T. After 3 washes with PBS, the cells were stained with a secondary antibody, HRP-conjugatedanti-Rabbit IgG (ZytoChem Plus HRP Polymer anti Rabbit, Zytomed, Berlin, Germany), then incubated with DAB (SuperPicture™ Polymer Detection Kit, DAB, rabbit, Thermo Fisher Scientific, MA, United States) for 15 min and then washed with distilled water. The cell cultures from both staining methods were captured by a digital camera (Olympus DP70, Olympus, Tokyo, Japan) with a calibration scale, 10 fields from each treatment by ×40 magnification were analyzed by shade using ImageJ software (NIH., Bethesda, MD, United States). The percentage of osteoclasts in the culture was calculated.

### 2.7 Statistical analysis

Statistical Packages for the Social Sciences (SPSS) or GraphPad Prism were used to analyze all experiments. Descriptive statistics, including means and standard deviation (SD), are shown for each data point. Comparisons between 2 groups were done using unpaired *t*-test and for more than 2 groups, using one-way ANOVA or mixed-designed ANOVA analysis. The level of statistical significance was set at 5%, and *p* values are indicated between treatments that showed statistically significant differences.

## 3 Results

### 3.1 Zoledronic acid and dexamethasone stimulate IFN-β production in macrophages

**FIGURE 1:**
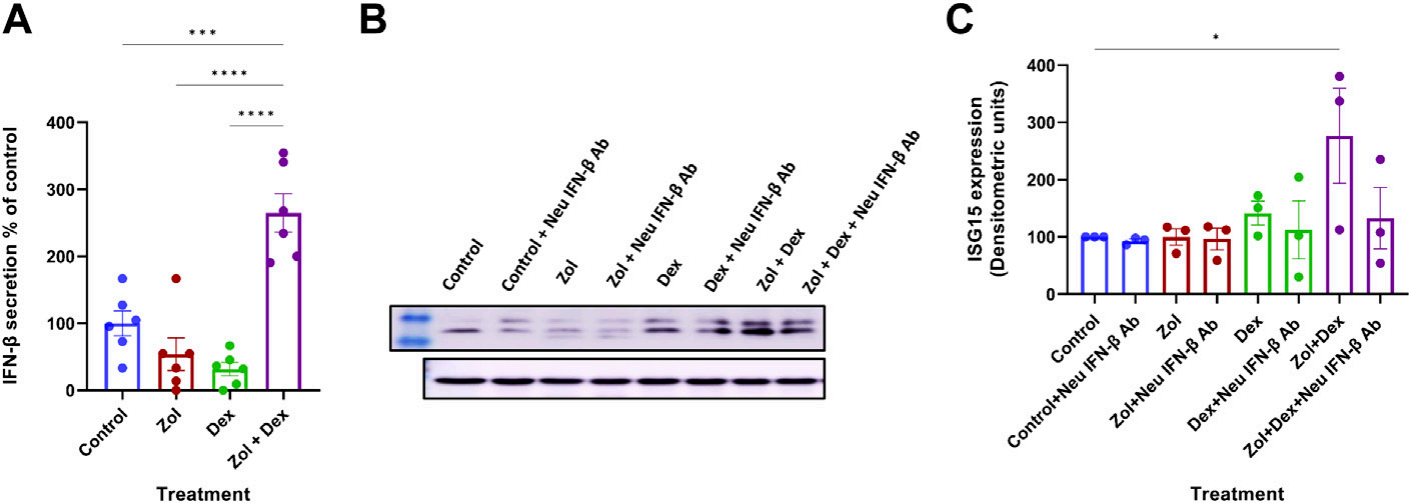
Zol+Dex promotes IFN-β secretion and ISG15 expression in RAW 264.7 macrophages. RAW 264.7 macrophages were incubated with Zol (10 µM) and/or Dex (1 µM) for 4 **(A)** or 24 h **(B,C)**. Then, culture medium was collected, and IFN-β levels were determined by standard ELISA (A,% C.V. were: Control = 45.73%, Zol = 110.6%, Dex = 76.80%, Zol+Dex = 26.41%), or cells were collected and analyzed by Western blotting for ISG15 and GAPDH **(B–C)**. Results are averages from 3 experiments **(A,C)** or representative images **(B)**. Statistical significance by one way ANOVA is indicated between the indicated treatments. *p < 0.05, ***p < 0.001, ****p < 0.0001.

Combined therapy using Zol+Dex has shown activity in MM. However, the synergy between the drugs leads to reduced skeletal-related events with unclear mechanisms (Tosi et al., 2006). We hypothesized that Zol+Dex treatment blocks osteoclast differentiation *via* changes in IFN-β levels. Therefore, we analyzed changes in IFN-β secretion from RAW 264.7 macrophages following treatment with Zol (10 µM), Dex (1 µM), and Zol+Dex or vehicle. After 4 h of incubation, IFN-β levels were evaluated by ELISA. The results showed the combined treatment with Zol and Dex for 4 h, but not with each drug alone, induced an increase in IFN-β secretion (Figure 1A). This regulation was specific for IFN-β as neither IFN-α nor IFN-γ transcription was upregulated by Zol+Dex (Supplementary Figure S1). Notably, the increase in IFN-β secretion was associated with a corresponding increase in the expression of ISG15 by macrophages exclusively following Zol+Dex treatment (Figures 1B,C). Thus, the combined treatment with Zol and Dex seems to induce the secretion of biologically-active IFN-β by macrophages.

### 3.2 RANKL induces osteoclastic differentiation of RAW 264.7 macrophages

**FIGURE 2:**
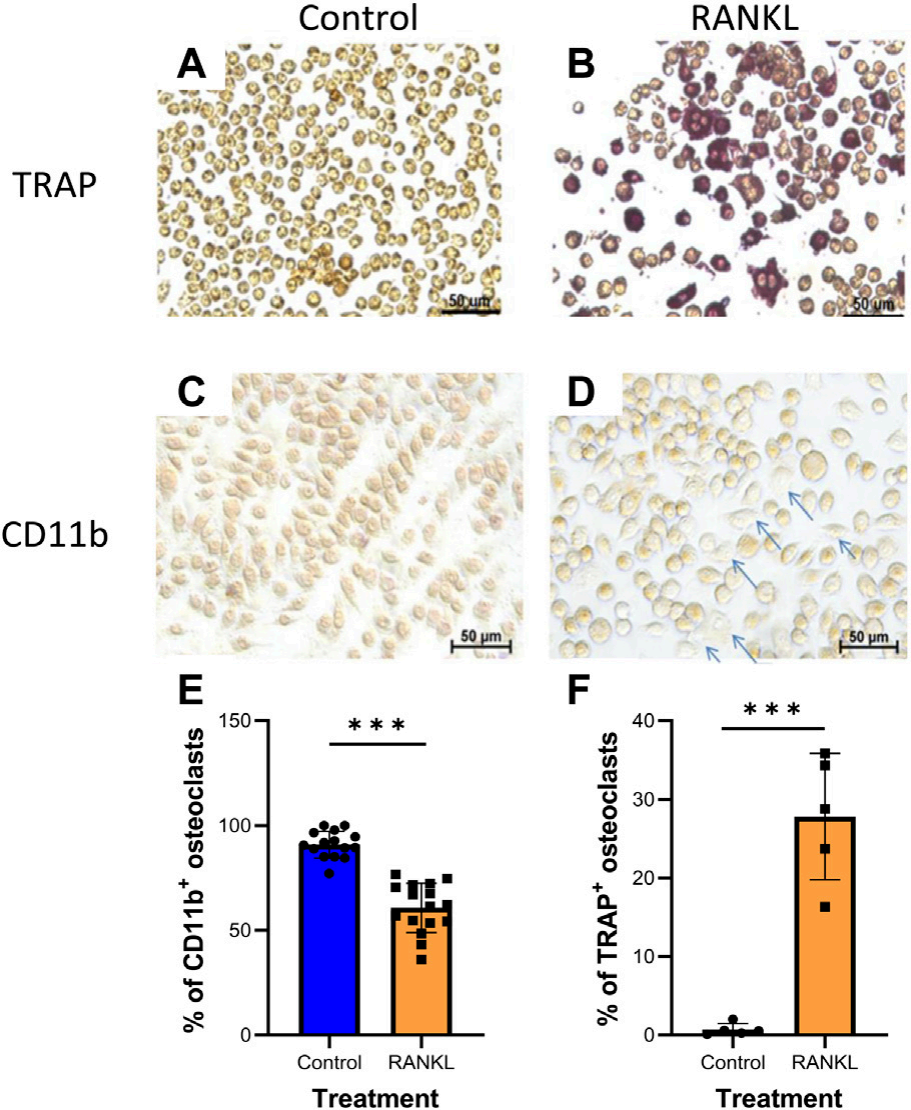
RANKL induces osteoclastogenesis in RAW 264.7 macrophages. RAW 264.7 cells were stained for TRAP **(A,B)**; *n* = 5 or CD11b **(C,D)**; *n* = 3 (arrows indicate unstained differentiated cells). **(E–F)** Averages of quantification of osteoclasts (TRAP^+^, CD11b^-^cells) using the ImageJ program. Statistical significance by Student’s *t*-test between control and RANKL treatments is indicated. ***p < 0.001.

Next, we determined the effect of Zol and/or Dex on osteoclastic differentiation of RAW 264.7 macrophages. To this end, we first determined whether RAW 264.7 macrophages differentiate into osteoclasts upon exposure to the osteoclastogenic cytokine RANKL as in (Kats et al., 2016). Two staining methods were used to identify the cells in the culture: 1) TRAP staining, which stains osteoclasts, and 2) CD11b staining, which identifies undifferentiated macrophages. Our results showed that treatment with RANKL (30 ng/ml) for 5 days resulted in macrophage differentiation to osteoclasts manifested by an increase in the TRAP^+^ cells (from 1.02 ± 0.67% to 30.7 ± 4.81% of the cells) and a concomitant decrease in CD11b^+^ cells (from 92.03 ± 5.1% to 60.1 ± 11.47% of cells). Overall, these results suggest that ∼30.7% of the macrophages differentiated into osteoclasts when cultured with RANKL. The differences between RANKL and control treatments were significant for both staining methods (P*** = 0.0001, Figure 2). Since these results indicate that both staining methods provide similar levels of osteoclastic differentiation, we exclusively used TRAP staining in the following experiments.

### 3.3 Zoledronic acid+dexamethasone and IFN-β reduce osteoclastic differentiation of macrophages and increase intracellular IFN-β

**FIGURE 3:**
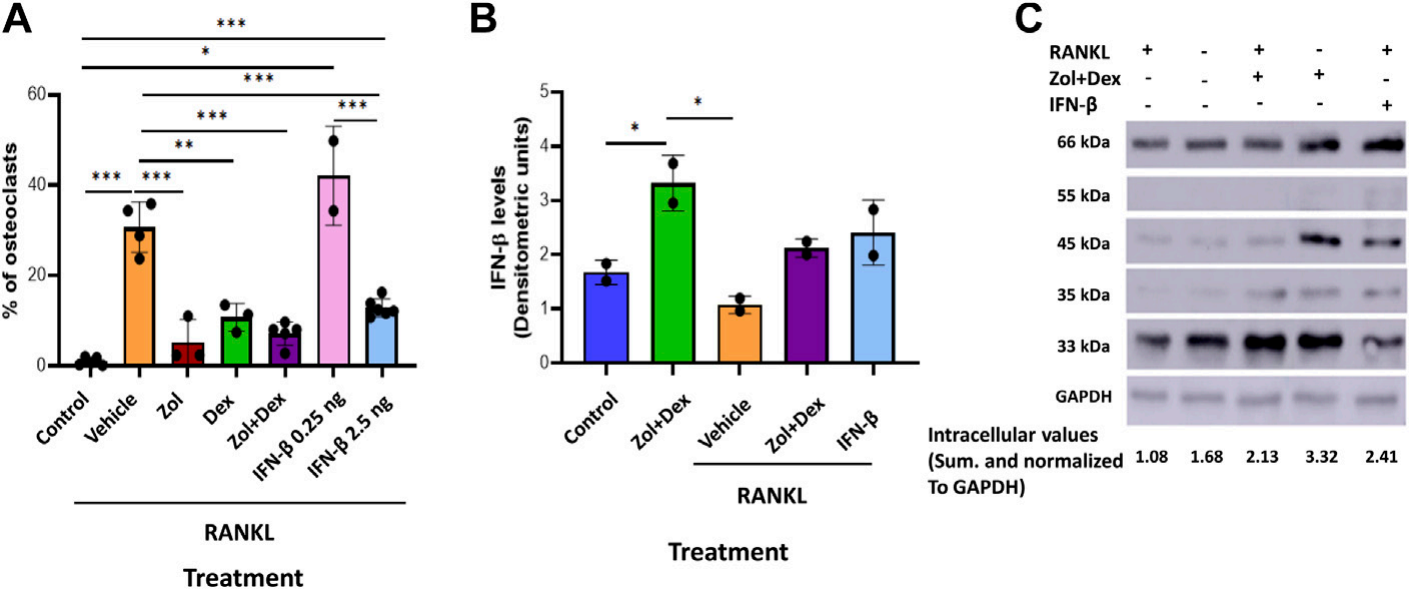
Zol+Dex and IFN-β inhibit RANKL-induced osteoclastogenesis. **(A)** Macrophages were treated with RANKL and Zol and/or Dex (10 µM each), or 0.25–2.5 ng/ml IFN-β for 48 h to induce osteoclast differentiation (after 5 days). Then, cells were fixed, stained for TRAP, and image analysis was performed using the ImageJ software. **(B)** Quantitative analysis of macrophages treated with or without RANKL, Zol+Dex, or IFN-β for 24 h. The cells were harvested, and W.B. for IFN-β was performed. The high molecular weight (more than 33 kDa) species of IFN-β underwent densitometric analysis, and the obtained values for each treatment were summed and normalized to GAPDH. **(C)** Representative IFN-β blotting image. Statistical significance by one-way ANOVA between matched treatments is indicated **(A–B)**. *p < 0.05, **p < 0.01, ***p < 0.001, ****p < 0.0001 (*n* = 2–5).

Our previous findings showed osteoclastic differentiation of 30.7% of macrophages when cultured with RANKL. Next, we determined the effect of Zol and/or Dex or IFN-β on osteoclastic differentiation. To this end, macrophages were cultured with RANKL for 5 days. In the first 48 h, the cells were supplemented with RANKL and Zol, and/or Dex (10 μM each) or IFN-β (0.25–2.5 ng/ml). After 5 days, the percentage of TRAP^+^ cells was quantified. As previously, 30.7% ± 4.81 of macrophages underwent osteoclastic differentiation (*n* = 5) when cultured with RANKL compared to 1.02 ± 0.67% in the control treatment (*p* < 0.0001). Treatment with Zol+Dex decreased osteoclastic differentiation to 7.12 ± 2.31% (*n* = 5, P** = 0.002 compared to RANKL + group). Moreover, treatment with Zol or Dex alone gave similar results (6.3 ± 4.1% and 7.8 ± 2.5% of cells, respectively; **p = 0.005 and *p = 0.04, respectively) to the Zol+Dex treatment. As expected, treatment with 2.5 ng/ml of IFN-β antibodies reduced osteoclastic differentiation to 13.5% and was statistically significant compared to RANKL alone or with 0.25 ng/ml IFN-β (***p = 0.007, **p = 0.006, respectively). Notably, Zol+Dex treatment decreased osteoclastic differentiation to a similar extent as IFN-β (Figure 3, *n* = 4). Next, we determined whether RANKL affects Zol+Dex-induced IFN-β production. Our results show IFN-β levels were reduced following RANKL exposure compared to control treatment. However, higher levels of IFN-β were found when macrophages were treated with Zol+Dex or IFN-β, and RANKL (2.13 ± 0.12 and 2.4 ± 0.42 DU, respectively; *p* < 0.05). In addition, Zol+Dex treatment without RANKL (3.32 ± 0.36 DU) resulted in the highest intracellular levels of IFN-β compared to controls (1.67 ± 0.16 DU). Overall, these results suggest that treatment of macrophages cultured with RANKL with Zol+Dex or IFN-β reduced osteoclastic differentiation and increased intracellular IFN-β levels.

### 3.4 IFN-β neutralization rescues osteoclastic differentiation of macrophages following Zoledronic acid+dexamethasone treatment

**FIGURE 4:**
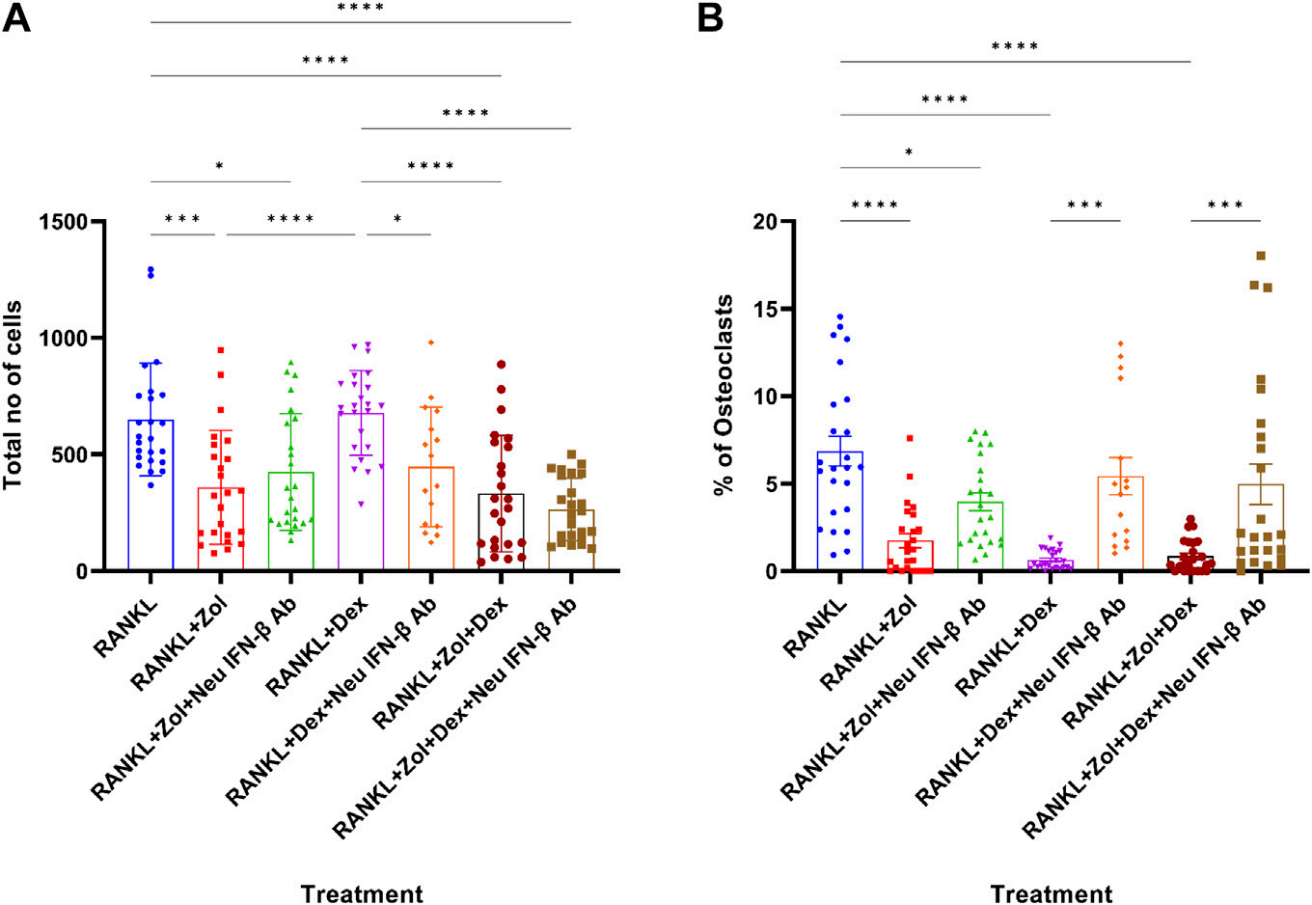
Drug-induced inhibition of osteoclastogenesis is mediated by IFN-β. Macrophages were treated for 48 h with RANKL and Zol, Dex or Zol+Dex, and anti-IFN-β neutralizing antibodies (2 μg/ml). Then, culture media was replaced and resupplemented with RANKL for additional 3 days. Next, the cells were stained for TRAP and enumerated, and analysis was performed by the ImageJ software for total cell number **(A)** and % of osteoclasts **(B)**. Statistical significance by one-way ANOVA is indicted (*n* = 3, 8 fields counted). *p < 0.05, **p < 0.01, ***p < 0.001, ****p < 0.0001.

Since Zol+Dex elevated IFN-β levels in RANKL-treated macrophages and IFN-β reduce osteoclastogenesis in these cells, we examined the role of IFN-β in Zol+Dex induced blockade of osteoclastic differentiation of macrophages. To this end, macrophages were treated with RANKL and Zol+Dex or IFN-β (2.5 ng/ml, as control) as well as anti-IFN-β neutralizing antibodies for 48 h. Then, the medium was replaced and resupplemented with RANKL. After additional 3 days, osteoclastic differentiation was measured by TRAP staining. Our results in Figure 4A indicate that Zol and Zol+Dex reduced macrophage numbers, whereas Dex did not. Notably, IFN-β neutralization did not affect Zol-induced cell death but did promote it in Dex-treated macrophages. Importantly, IFN-β neutralization also significantly restored osteoclastic differentiation following Dex or Zol+Dex treatment (***p < 0.001) but not following Zol alone (Figure 4B). As expected, treatment with anti-IFN-β antibodies did not affect RANKL-induced osteoclastogenesis (data not shown). Notably, neither STAT1 nor STAT3 inhibition reversed the anti-osteoclastogenic actions of IFN-β or Zol+Dex (Supplementary Figure S2), suggesting that other STAT family members mediate the activity of the Zol+Dex-IFN-β axis. Thus, the abrogation of osteoclast differentiation from macrophages induced by Zol+Dex is mediated, at least in part, by early production of IFN-β.

### 3.5 Zoledronic acid and dexamethasone induce IFN-β expression by resolution phase macrophages

**FIGURE 5:**
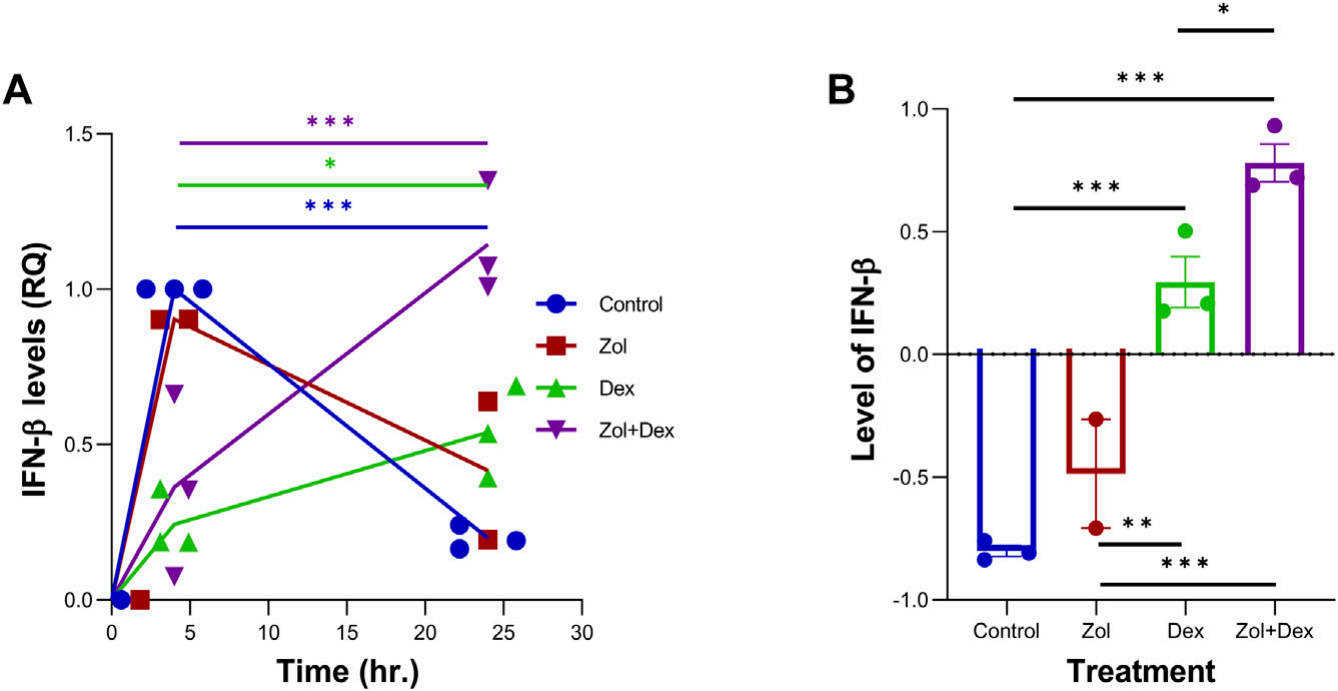
Zol+Dex increases IFN-β transcripts in peritoneal macrophages at 24 h. Male C57BL/6 mice were injected intraperitoneally with zymosan A (1 mg/mouse). After 66 h, the peritoneal exudates were collected, and peritoneal macrophages were isolated. Resolution phase peritoneal macrophages were collected and used immediately (time 0) or incubated for 4 or 24 h with Zol, Dex, or Zol+Dex, 10 µM each. R.N.A. was isolated from the samples, and RT-PCR for IFN-β was performed **(A)** Quantitative analysis of RT-PCR assay of IFN-β transcripts, comparison between 4 and 24 h **(B)** Differences between 24 and 4 h of each treatment. Statistical significance by one-way ANOVA (*n* = 9). *p < 0.05, **p < 0.01, ***p < 0.001.

Dex was previously shown to promote macrophages conversion to the pro-resolving satiated/CD11b^low^ phenotype and enhance IL-10 production by these cells (Schif-Zuck et al., 2011), whereas IFN-β was shown to promote the same events (Kumaran Satyanarayanan et al., 2019). Therefore, we sought to determine whether Dex and/or Zol can promote IFN-β expression in resolution phase macrophages. To this end, we recovered macrophages 66 h post zymosan A-induced peritonitis and cultured them for 4–24 h with the indicated drugs. Our results in Figure 5 show a robust increase in IFN-β expression in vehicle and Zol treatments that significantly declined at 24 h. Dex and, to a higher degree, the Zol+Dex treatment induced a much lesser induction of IFN-β at 4 h, but this response ascended at 24 h. Thus, Dex seems to induce IFN-β production by resolution phase macrophages, which is enhanced by treatment with Zol.

## 4 Discussion

Skeletal-related events are a common complication of hematological malignancies and cause severe pain, increased risk of death, and reduced quality of life. The impact of zoledronic acid in the prevention of pain and bone fractures in MM was confirmed in a meta-analysis that evaluated 20 randomized clinical trials with nearly 7,000 patients (Alegre et al., 2014). The direct suppression of osteoclast function by BPs and its consequences on bone remodeling has been reported in a few *in vivo* studies (Sharma et al., 2013; Alvarez et al., 2019). These effects are perceived to be caused by the inhibition of the intracellular mevalonate (Mev) pathway and the loss of farnesyl pyrophosphate (FPP) and geranygeranyl pyrophosphate (GGPP) synthesis (Gibbs and Oliff, 1997). Glucocorticoids, such as dexamethasone, play an important role in MM treatment. While glucocorticoids have single-agent activity in MM, their combination with other drugs induces higher clinical responses (Burwick and Sharma, 2019). Here, we investigated a potentially new mechanism of action for combined therapy with BPs and Dex in limiting bone resorption, a likely basis for medication-related osteonecrosis of the jaw. Our results showed that the combination of Zol and Dex increased IFN-β secretion as well as the expression of ISG15. We also found that treatment with Dex, Zol+Dex, or IFN-β alone limited osteoclastogenesis in an IFN-β-dependent manner, irrespective of STAT1 or STAT3 activation.

Dex has been previously shown to limit inflammation and promote its resolution by limiting neutrophil accumulation (Perretti et al., 2002) and enhancing apoptosis of inflammatory (M1) macrophages while promoting the survival of anti-inflammatory macrophages through the adenosine A3 receptor (Barczyk et al., 2010; Achuthan et al., 2018). Dex was also found to enhance the ability of macrophages to engulf apoptotic cells, a key event in the resolution of inflammation (Maderna et al., 2005). In murine peritonitis, Dex was found to promote the uptake of apoptotic cells and limit inflammatory cytokine production while enhancing IL-10 secretion (Schif-Zuck et al., 2011). Notably, we have recently shown elevated levels of IFN-β in peritoneal exudates during the resolution phase of peritonitis and pneumonia in mice, particularly following the uptake of apoptotic cells by resolution-phase macrophages (Kumaran Satyanarayanan et al., 2019). IFN-β, in turn, promotes macrophage efferocytosis and reprogramming to anti-inflammatory phenotypes (Kumaran Satyanarayanan et al., 2019). Thus, we hypothesized that Dex alone or combined with Zol would induce IFN-β expression and secretion from macrophages. Unexpectedly, our results (Figure 1A) showed that only the combination of Zol+Dex, and not each drug alone, induced a rapid secretion of IFN-β. This secretion did not sustain through 24 h (data not shown). However, it was sufficient to result in a significant increase in the expression of the IFN-β triggered gene ISG15 in Zol+Dex treated macrophages (Figures 1B,C). The fast secretion of IFN-β upon treatment with both drugs suggests that this response does not involve the uptake of apoptotic macrophages but rather the rapid release of internal stores of IFN-β, and could be a result of drug interaction. Thus, Zol and Dex induce a biologically active form of IFN-β from RAW 264.7 macrophages.

Recent publications have shown that type I IFNs decreased Mev lipid synthesis during inflammation (York et al., 2015) and inhibited osteoclast differentiation (Takayanagi et al., 2002). Notably, it was previously shown that BPs induce high levels of IFN-β in osteoclasts, which in turn promotes osteoblast maturation and bone formation (Ma et al., 2018). Moreover, Type I IFN signaling was recently found to limit age-related bone loss and osteoclastogenesis through the induction of guanylate-binding protein (GBP) 5 (Ho and Ivashkiv, 2006; Place et al., 2021). In the current study we have shown only the combined treatment with Zol + Dex, but not with each compound alone, increased IFN-β secretion and the expression of ISG15 in an IFN-β dependent manner in RAW 264.7 macrophages. Moreover, IFN-β, at low concentrations (2.5 ng/ml) inhibited macrophage differentiation to osteoclasts, and IFN-β blockage significantly abrogated either Dex or Zol+Dex inhibition of osteoclastogenesis but did not affect the control treatment. Altogether, these results support our hypothesis that Zol+Dex block macrophage osteoclastogenesis through the secretion of IFN-β and its action on macrophages that might also involve attenuation of Mev synthesis.

In macrophages, IFN-β activates signal transducers and activators of transcription (STAT) 1 and STAT3, mediating the antiviral and inflammatory effects of IFN-β (Ho and Ivashkiv, 2006; Kumaran Satyanarayanan et al., 2019). To detect whether STAT1 or STAT3 mediates the inhibitory effect of Zol+Dex or IFN-β on osteoclastogenesis, specific inhibitors of these transcription factors were used in the aforementioned differentiation assay. Our results indicate that neither the STAT1 nor the STAT3 inhibitor restored the differentiation of osteoclasts upon inhibition by Zol+Dex (Supplementary Figure S2). Nevertheless, the STAT1, but not the STAT3 inhibitor, restored osteoclastogenesis (47.18% recovery) upon inhibition by IFN-β. Notably, this recovery did not reach statistical significance, probably due to the low concentration of fludarabine. Recent publications have shown that STAT3 inhibitors down-regulate the expression of T-bet, GATA3, IL12Rb2, and IFN-γ, as well as the formation of osteoclasts (Holland et al., 2007; Li, 2013). On the other hand, another report has shown that STAT3 deficiency causes skeletal and connective tissue disorders. Notably, Zol treatment increases bone density in these patients by inhibiting the protein suppressor of cytokine signaling 3 (SOCS3), which results in a switch from IL-6 to IL-10 production in macrophages and a decrease in bone loss. The transcription of SOCS3 is regulated by nuclear accumulation of phosphorylated STAT3, and STAT3 is downregulated by SOCS3 (Staines Boone et al., 2016). These results support our conclusion that inhibition of STAT3 does not promote osteoclast differentiation.

STAT1 is essential for gene activation in response to interferon stimulation. Recent publications showed high levels of osteoclasts in bone marrow macrophages from STAT1-deficient mice treated with IFN-β and RANKL (Takayanagi et al., 2002). This manuscript has suggested a signaling cross-talk between RANKL and IFN-β *via* ISGF3, which is composed of STAT1, 2, and IRF-9, and that inhibition of STAT1 impairs the osteogenesis processes by enhancing osteoclast differentiation. Another publication showed that STAT1 protein levels decreased over time after Zol treatment (Muratsu et al., 2013). Our results have shown that STAT1 inhibition did not affect the drug treatment but partially restored osteoclastogenesis upon treatment with IFN-β, albeit without statistical significance. These results suggest STAT1 is not involved in drug-induced IFN-β expression. However, the inhibitory effect of IFN-β on osteoclastogenesis might be dependent, at least in part, on STAT1 activity.

The bone destruction in MM is mediated by osteoclasts, specialized bone-resorbing cells engaged in normal bone remodeling. Myeloma cells and marrow stromal cells produce factors that induce osteoclast formation and activation, thus changing the balance between bone apposition and bone resorption (Muratsu et al., 2013). Combinational therapy of Zol+Dex is clinically effective in preventing and managing myeloma-induced bone disease (Mhaskar et al., 2012). Additional osteoclasts targeting drugs such as: Cyclosporin A (Orcel et al., 1991), Revoremycin A, RANKL antibodies (Ding et al., 2021), Idelalisib (Yeon et al., 2019) and Compactin (Woo et al., 2000) were found to inhibit different satges in osteoclastogenesis and may be usefull to treat MM.

Our findings suggest a new pathway for suppressing bone resorption that involves IFN-β. Within the limits of this study, it can be concluded that Zol+Dex promotes IFN-β secretion. Consequently, IFN-β limits macrophage differentiation into osteoclasts downstream of the Zol+Dex treatment. Notably, IFN-β based therapies are used to treat multiple sclerosis patients with no evidence of MRONJ (Jongen et al., 2011). Thus, IFN-β therapy might be used to inhibit osteoclastogenesis in MM patients, with minimal risk to develop osteonecrosis of the jaw.

## Data Availability

The original contributions presented in the study are included in the article/Supplementary Material, further inquiries can be directed to the corresponding author.
