# Supplementary figures and images for: IFN-β mediates the anti-osteoclastic effect of bisphosphonates and dexamethasone
Source: Front Pharmacol. 2022 Oct 14;13:1002550. doi: 10.3389/fphar.2022.1002550 (PMC9648992; doi:10.3389/fphar.2022.1002550)

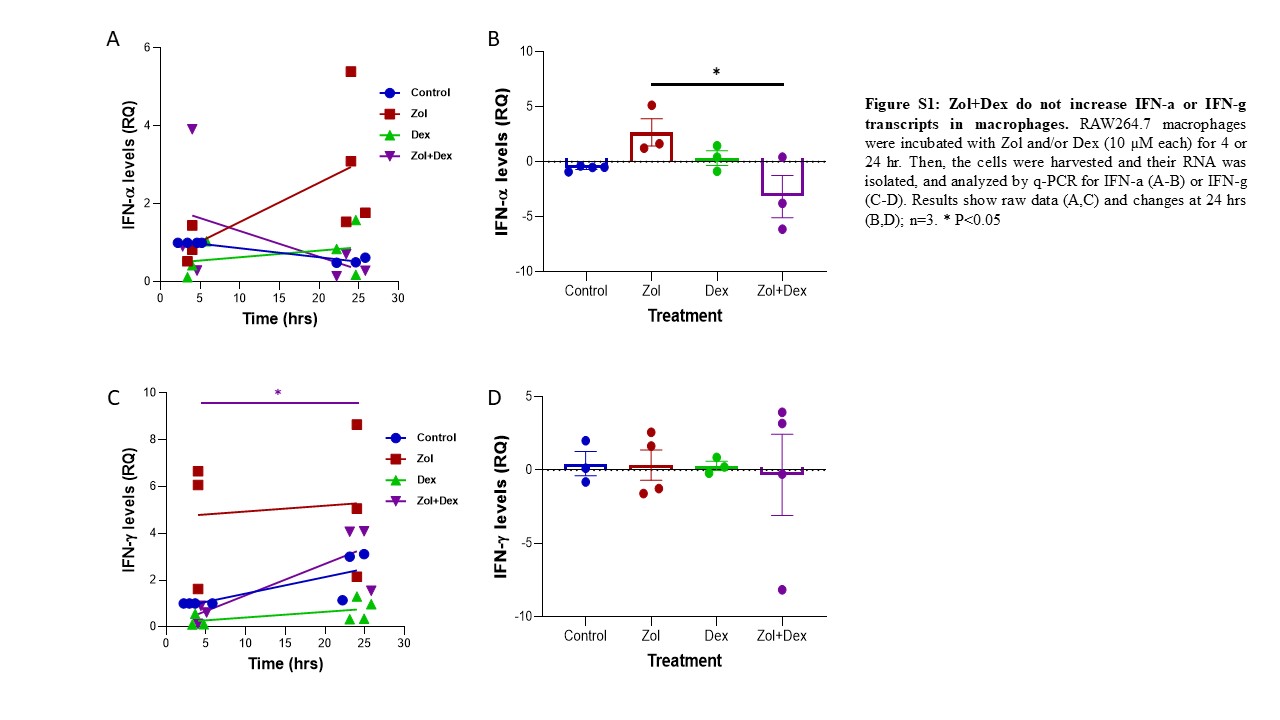

Supplement: Supplementary file 1 [file Image1.JPEG]

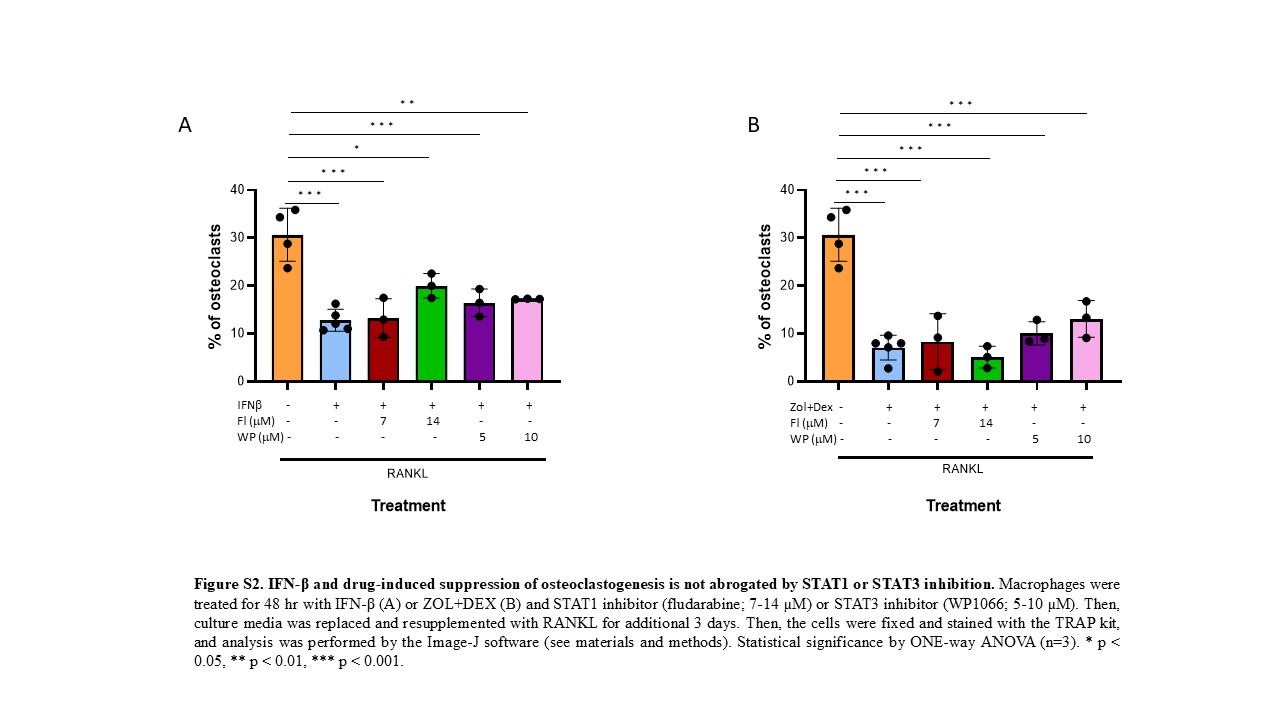

Supplement: Supplementary file 2 [file Image2.JPEG]
